# Rub-Impact Fault Diagnosis Using an Effective IMF Selection Technique in Ensemble Empirical Mode Decomposition and Hybrid Feature Models

**DOI:** 10.3390/s18072040

**Published:** 2018-06-26

**Authors:** Alexander E. Prosvirin, Manjurul Islam, Jaeyoung Kim, Jong-Myon Kim

**Affiliations:** School of Electrical, Electronics and Computer Engineering, University of Ulsan, Ulsan 44610, Korea; a.prosvirin@hotmail.com (A.E.P.); m.m.manjurul@gmail.com (M.I.); kjy7097@gmail.com (J.K.)

**Keywords:** degree-of-presence ratio, ensemble empirical mode decomposition, fault diagnosis, feature extraction, intrinsic mode function selection, Kullback–Leibler divergence, rotating machinery, rub-impact faults

## Abstract

The complex nature of rubbing faults makes it difficult to use traditional signal analysis methods for feature extraction. Various time-frequency analysis approaches based on signal decomposition, such as empirical mode decomposition (EMD) and ensemble EMD (EEMD), have been widely utilized recently to analyze rub-impact faults. However, traditional EMD suffers from “mode-mixing”, and in both EMD and EEMD the relevance of the extracted components to rubbing processes must be determined. In this paper, we introduce a new informative intrinsic mode function (IMF) selection method for EEMD and a hybrid feature model for diagnosing rub-impact faults of various intensities. Our method uses a novel selection procedure that combines the degree-of-presence ratio of rub impact and a Kullback–Leibler divergence-based similarity measure into an IMF quality metric with adaptive threshold-based selection to pick the meaningful signal-dominant modes. Signals reconstructed using the selected IMFs contained explicit information about the rubbing faults and are used for hybrid feature extraction. Experimental results demonstrated that the proposed approach effectively defines meaningful IMFs for rubbing processes, and the presented hybrid feature model allows for the classification of rub-impact faults of various intensities with good accuracy.

## 1. Introduction

Rotating machines, such as turbines, are widely used for power generation and usually operate under severe operating conditions characterized by high temperatures and high rotational speeds. The goal of the turbine design process is to maintain a small clearance between the rotor blades and the stator to increase torque and reduce air reluctance. Rubbing occurs when the turbine blades interact with the stationary parts, either due to blade expansion resulting from high temperatures or due to faults, such as misalignment of the shaft and self-excited vibrations [1]. If faults are not detected at an early stage, rubbing can cause excessive damage to the rotating machine, significantly increasing the maintenance cost. Therefore, the purpose of rub-impact fault diagnosis is to extract intrinsic information about rub impact and apply the extracted features to a classifier to determine rubbing faults at different intensities.

Rubbing faults are recognized as highly complex nonlinear and nonstationary faults [2,3], which cause a large number of transients to appear in the signal. This makes it difficult to utilize traditional signal-processing techniques for feature extraction, such as time-domain and frequency-domain fast Fourier transform (FFT) analysis, which cannot efficiently detect transient phenomena in a nonstationary rub-impact signal due to the inherent constraints of these methods. Therefore, the features extracted from rubbing signals by these methods may not be informative and may not reflect the actual rubbing processes but rather the qualities of a particular test rig and testing environment. In general, the quality of such features is not high enough to differentiate rub-impact faults of different intensities.

To overcome the nonlinearity and nonstationarity constraints in rubbing fault feature extraction and diagnosis, many techniques based on time-frequency domain analysis (TFA) have been introduced over the last decade [4,5,6,7,8,9,10,11,12,13,14]. Yanjun et al. [4] proposed wavelet packet eigenvalue calculation as a feature extraction technique for rubbing fault diagnosis. In this method, wavelet packet analysis is used to improve the time-frequency resolution of the vibration signal. In addition, the maximal singular values (SVs) of the coefficient matrix obtained from wavelet packet decomposition are used as feature vectors for a support vector machine (SVM) classifier. Though the features proposed by these authors demonstrated good accuracy, the method may not be appropriate for early-stage rubbing fault diagnosis because the normal system conditions and different rubbing intensities were not considered in their experiments. Zhihao et al. [5] suggested the use of discrete wavelet transformation (DWT) and time-domain signal extraction at level 3 of decomposition as a feature extraction approach for identifying rubbing faults. To classify signal samples, this extracted datum of the third level was used as the input to the SVM model. Although the authors claim that the rate of correct rubbing fault identification was more than 92%, this study did not consider rub-impact faults of different intensities and did not suggest any techniques for adaptive level or sub-band selection to ensure that the presence of a rubbing fault in the selected datum is significant. Moreover, the use of time-domain data as a feature vector for SVM may not be suitable for situations when the acquired signals include a large number of time points, even when these signals are supplemented by down-sampling properties of wavelet transformation. Roy et al. conducted a study on the efficacy of various wavelet mother functions and signal-filtering techniques for rub-impact fault detection [6]. This study emphasized the importance of finding the proper combination of wavelet mother functions and filtering techniques, both of which strongly affect the accuracy of rub-impact fault detection. Similar limitations of the wavelet transform were reported by other researchers [7]. It is usually not easy to choose the optimal wavelet function for a complex and nonlinear signal, and since the process of determining a proper wavelet function requires a series of experiments, one can conclude that some subjectivity is present in this process [8,9]. Deng et al. [10] introduced a combination of local mean decomposition (LMD) and Teager energy kurtosis (TEK) as a feature extraction technique for rub-impact fault diagnosis. This approach allowed for the extraction of numerical features from faulty signals and was capable of distinguishing rub-impact faults. However, it did not consider various rubbing intensities. In addition, the TEK values for the sixth and seventh product functions (PFs), which corresponded to the 1/3× fractional frequencies in their study, were not distinguishable between normal and fault conditions even though these fractional frequencies are usually recognized as critical indicators of the rubbing process [11,12]. Yang et al. [13] proposed an ensemble LMD (ELMD) that improves the ability of LMD to decompose rub-impact signals by calculating the ensemble mean of extracted PFs. This study mostly contributed to the field of signal processing in the areas of rubbing fault detection and the observation of a rubbing phenomenon over the frequency spectrum of the extracted PFs; no numerical features that could be used to differentiate between types and intensities of rubbing faults were considered in the experimental part. Another study [14] proposed the nonlinear squeezing time-frequency transform (NSTFT) as a TFA approach for rub-impact detection. This study simulated various rub-impact models and analyzed them using the proposed method, providing detailed information about frequency harmonics and the behavior of instantaneous frequencies when a rubbing fault occurs in a system. However, the focus of the study was the detection of rubbing faults, and no experiments were performed on rubbing faults with varying intensities.

Recently, a state-of-the-art TFA approach called empirical mode decomposition (EMD), introduced by Huang et al. [15], has become widely used for fault diagnosis in rotating machinery, such as rotors, bearings, and gears [16]. Moreover, EMD appeared to be an efficient decomposition technique that can be used in other applications [17,18,19]. EMD itself is a very powerful and convenient algorithm that allows for the analysis of complex and nonlinear multicomponent signals by decomposing them into a finite number of intrinsic mode functions (IMFs). One of the most important advantages of EMD over its counterparts is that it is an entirely data-driven and self-adaptive signal decomposition technique in which each IMF corresponds to a natural oscillatory component of the original signal and can be treated as a specific frequency sub-band. Due to these advantages, EMD has been successfully used to extract and observe discriminative information from rub-impact fault signals in several studies [20,21,22,23]. These studies provide a good insight into the capability of EMD and its IMFs to extract rub-impact information from the vibration signal of rotor systems. However, they did not perform any feature extraction, instead focusing on the visual observation of rubbing faults. Yibo et al. [24] proposed calculating the maximum SVs from each of the extracted IMFs to create feature vectors for classifying rub-impact fault models using SVM. This feature extraction approach allows for differentiation between different rub-impact faults types; however, the efficacy of the proposed features in differentiating normal system conditions when there is no rubbing and various rubbing intensities was not investigated. This approach also ignores the process of selecting meaningful IMFs.

Despite its advantages over its main counterpart, wavelet family decomposition, the traditional EMD algorithm has shortcomings that may cause problems when it is used for feature extraction to differentiate machinery faults. Specifically, the conventional EMD technique can result in abnormalities, such as the presence of either disparate oscillations in one IMF or very similar oscillations in different IMFs. This is called the mode-mixing problem and makes it difficult to obtain a clear physical interpretation of each IMF, which is essential for fault feature extraction and diagnosis. Several variants of EMD have therefore been introduced to resolve the mode-mixing problem and improve the traditional approach. The breakthrough in EMD improvement was a method called ensemble empirical mode decomposition (EEMD) [16,25]. This method is reported to successfully resolve the problem of mode-mixing by performing EMD over an ensemble of the signal with the addition of white Gaussian noise. EEMD decomposition has already been successfully used for rolling-element bearing [26,27] and rub-impact fault diagnosis [28]. Regarding rub-impact faults, Lei et al. [28] utilized EEMD to diagnose rubbing faults in a power generator and early rub-impact faults in a heavy oil catalytic cracking machine set. These experimental results demonstrated the advantages of EEMD in extracting fault characteristics, even for incipient faults in rotating machinery.

Although EEMD successfully resolved the problem of mode-mixing, one issue that makes it difficult to utilize this approach for fault diagnosis is the adaptive determination of meaningful IMFs that contain specific and valuable information about the mechanical fault. As an EMD-based method, EEMD yields a finite set of IMFs after completing the decomposition process, and the cardinality of the extracted components is very large [29,30]. In practice, however, the extracted IMFs are not all equally important for fault feature extraction, and they can be either noise-dominant or signal-dominant. Therefore, it is necessary to select a set of discriminative IMFs in which each IMF is highly useful for fault feature extraction and carries essential information about the mechanical fault. Yi et al. [31] proposed the use of steady-state indexes for condition monitoring of railway axle bearings, where they also addressed the problem of selecting the meaningful IMF components obtained by EEMD by picking the single mode with the largest energy. This study demonstrates that the introduced steady-state indices extracted from the single selected IMF are robust features for axle bearing condition monitoring. However, the absence of the adaptive thresholding approach while selecting IMFs based on the energy might be an issue when applying this method for a rubbing fault problem where the relation “energy-content” for the extracted modes might not be that well-observable as in the investigated bearing faults.

In this literature review, we observed that the main directions of current studies are the detection of rubbing faults using different TFA methods, the representation of rubbing faults using various forms of frequency spectra, and the investigation of rubbing itself as a physical phenomenon. Almost no studies have focused on diagnosing rubbing faults of various intensities, and very few actual feature extraction models with numerical values that can be directly applied to classification techniques for rub-impact fault diagnosis have been proposed. Therefore, this paper proposes a reliable rub-impact fault feature extraction approach that combines EEMD with a new IMF selection procedure and uses the selected components to reconstruct the signal and perform hybrid feature extraction. The signal reconstructed using these selected informative IMFs contains less noise and clear rubbing fault frequency components, which makes the rubbing process easily observable. The proposed hybrid feature model for fault feature extraction allows for the examination of rubbing phenomena in both time and frequency domains and produces well-separable features that can be used for fault diagnosis. It is important to mention that there exist other metrics that can be used to quantify various repetitive transients and select informative portions (or sub-bands) of a signal, such as a kurtosis and its modification: a spectral kurtosis. These metrics were used in combination with other TFA decomposition techniques; however, these metrics appeared to be not exactly proportional to the degree of defectiveness of the system and the result highly depends on the frequency resolution assigned prior to the decomposition process [32,33].

The main contributions of this manuscript are as follows:

1. This paper presents a new informative IMF selection procedure that can be used to select the modes obtained by EEMD in rubbing fault analysis. The proposed method includes a new quality criterion for mode evaluation that combines the degree-of-presence ratio (DPR) of rub impact and the Kullback–Leibler divergence (KLD), a statistical similarity metric [34]. An adaptive selection technique then utilizes a thresholding approach and the aforementioned criterion to adaptively select the most valuable IMFs. The selected informative signal-dominant IMFs carry intrinsic information about the important harmonics of rub-impact faults.
2. Since the selected IMFs are highly effective at detecting rub-impact phenomena, this paper then extracts features for rub-impact fault diagnosis from the signal reconstructed using the selected components. Thus, a hybrid feature model that well-represents rub-impact fault conditions is presented in this study. Our hybrid feature model consists of four features directly extracted from the reconstructed signal in the time domain and three features extracted from the envelope power spectrum of this signal. This hybrid set of features is highly effective for representing each rub-impact fault condition, so these features are further used in a classifier for diagnosing rubbing faults with various intensities.

The remaining sections of this paper are structured as follows. Section 2 presents the proposed rub-impact fault feature extraction methodology, including the adaptive IMF selection procedure and hybrid feature model for diagnosing rubbing faults of various intensities. Section 3 provides experimental validation of the proposed methods. Finally, Section 4 contains the concluding remarks.

## 2. Proposed Rub-Impact Fault Feature Extraction Technique

**Figure 1:**
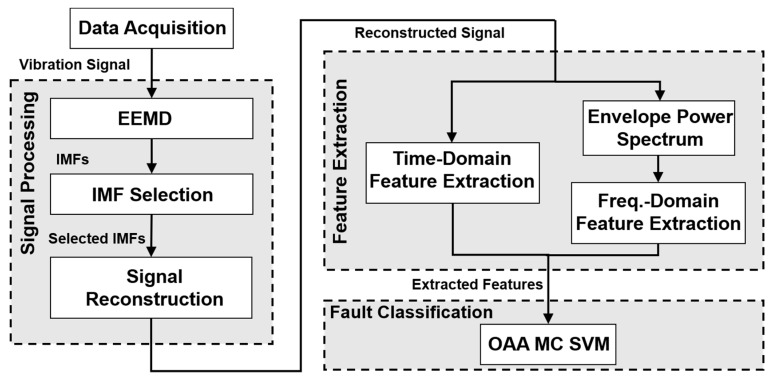
A block diagram of the proposed fault diagnosis framework including informative IMF selection, fault feature extraction, and one-against-all multi-class support vector machine (OAA MC SVM)-based fault classification. EEMD, ensemble empirical mode decomposition; IMF, intrinsic mode function.

The block diagram of the proposed rub-impact fault diagnosis framework is presented in Figure 1. The proposed approach consists of four essential steps: data acquisition, signal processing, feature extraction, and fault classification. As shown in Figure 1, after data acquisition, an unknown vibration signal is first decomposed into a finite number of oscillating components by EEMD. Then, the subset of IMFs that are signal-dominant and carry essential information about the rubbing faults is selected using the proposed adaptive selection procedure with our novel IMF evaluation metric. Next, time- and frequency-domain features are extracted from the signal reconstructed using the selected IMFs, which represents a clear rub-impact fault signal. This set of hybrid features can provide sufficient insight into the rubbing process to classify rubbing faults of various intensities. The fault diagnosis procedure is completed by means of a one-against-all multi-class support vector machine (OAA MC SVM) classifier [35,36].

### 2.1. Data Acquisition

**Figure 2:**
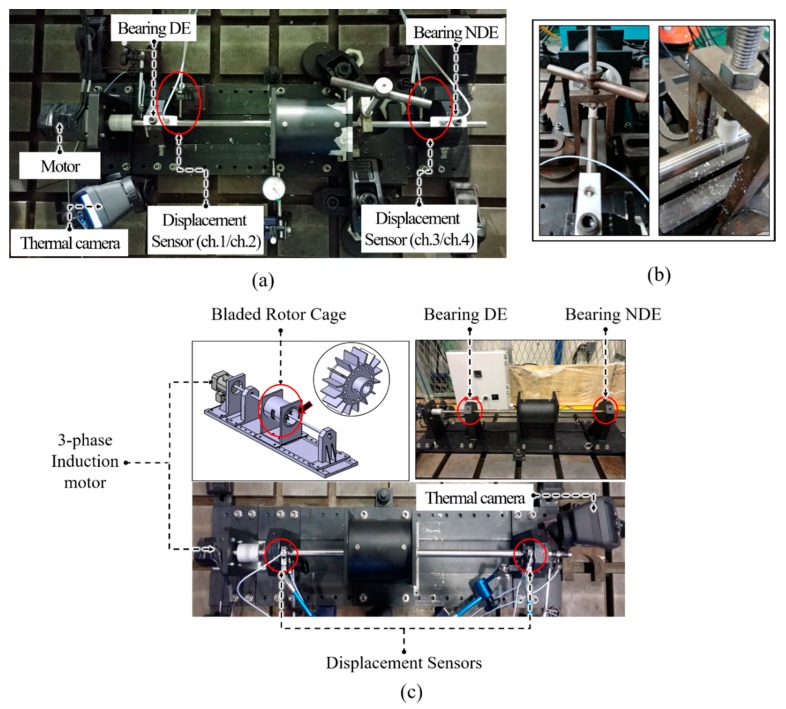
The self-designed test rig for rub-impact fault diagnosis: (**a**) the overall view of the testbed; (**b**) the device used to create shaft imbalance; and (**c**) simplified component view. DE, drive end; NDE, non-drive end.

Figure 2 shows the self-designed experimental testbed used to collect rubbing fault data by simulating rub-impact faults of different intensities. Two sensors were installed at the drive end (DE) and non-drive end (NDE) of the shaft to continuously measure vibrations of the rotor. Each sensor records the displacements of the 16-blade rotor in both the vertical and horizontal directions using a different channel for each. Therefore, a total of four channels were used by two vibration sensors to record displacements in the vertical and horizontal directions at both ends of the shaft. A Pulse 3560 C device was used to digitize the acquired signal. Details of the data collection system are provided in Table 1. The experiment was performed at a constant rotational speed of 2580 revolutions per minute (RPM), and the signal was sampled at a rate of 65.5 kHz.

**Figure 3:**
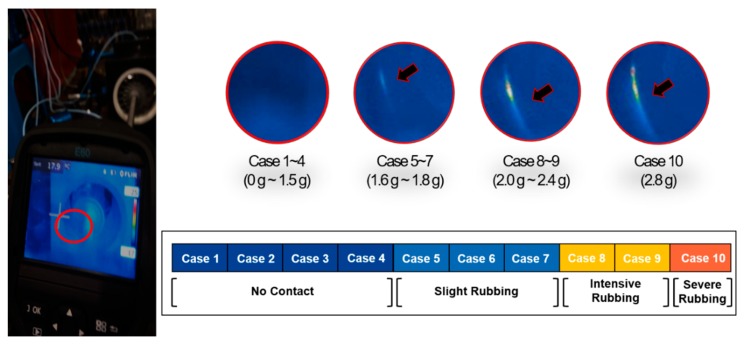
Changes in rubbing intensity validated using a thermal camera.

A rub-impact fault was simulated by adding extra weight on the NDE to create a shaft imbalance. This imbalance in the shaft caused local interactions between the rotor blades and the stationary part. The appearance and intensity of the rubbing process were validated using a thermal camera mounted on the NDE of the shaft. Adjusting the extra mass at the end of the shaft resulted in various rubbing intensities as shown in Figure 3. The total duration of the recorded signal for each case was 59 s. However, each signal was divided into slices of 1 s each for signal processing and feature extraction.

### 2.2. Empirical Mode Decomposition and Its Variant

In this subsection, we provide the necessary background on the original EMD and EEMD algorithms.

#### 2.2.1. Empirical Mode Decomposition

EMD [15] decomposes the original signal x(t) into a finite number of oscillatory components using a sifting process. In order to be defined as an IMF, the function should meet the following two conditions [16,37]: (i) the number of extrema and zero-intersect points are either equal to each other or differ by at most one; and (ii) at any point, the mean of the envelopes defined by local maxima and minima is equal to zero. Each IMF can be considered as a specific frequency band of the original signal, where the first IMFs represent high-frequency bands and the last IMFs correspond to lower frequency bands [15].

After the decomposition process, the input signal x(t) can be defined as shown below:

(1) $$x(t)=\sum_{i=1}^{n}{\mathrm{IMF}}_{i}(t)+r_{n}(t),$$

where n is the total number of extracted IMFs and $r_{n}\left(t\right)$ is the residue of the signal decomposition. The quality of the decomposition can be evaluated by computing the amplitude error between the original signal and the reconstructed one.

EMD is a powerful tool that is capable of extracting nonlinear and nonstationary parts of the original signal. However, its crucial disadvantage is the mode-mixing problem, which leads to multiple oscillating components being presented in a single IMF or similar oscillating components being split in different modes with smaller amplitudes. This drawback causes difficulties in interpreting the physical meaning of each mode for feature extraction and fault diagnosis.

#### 2.2.2. Ensemble Empirical Mode Decomposition

EEMD [16,25] was introduced to overcome the problems of mode-mixing observed in conventional EMD. The concept of EEMD is to obtain precise IMF components by taking the mean of several EMD trials performed on the original signal, with the addition of various realizations of white noise in each trial. The main advantage of EEMD over conventional EMD is that it significantly reduces the chance of mode-mixing and is capable of decomposing the original signal more precisely into a set of “true” IMFs. The implementation steps of EEMD are summarized as Algorithm 1.


**Algorithm 1: EEMD Algorithm**

Generate an artificial observation $x^{\;j}\;[n]=x\;[n]+\omega^{\;j}\;[n]$, where $\omega^{\;j}\;[n]\;(j=1,\ldots ,J)$ are various realizations of Gaussian noise.Using traditional EMD, completely decompose the ensemble $x^{\;j}\;[n]$ with added white Gaussian noise into IMFs, ${\mathrm{IMF}}_{\;k}^{\;j}\;[n]$. Here, k=1,…,K indicates the modes.Repeat steps 1 and 2 with different white noise realizations each time.Obtain the result of decomposition as an ensemble means of the corresponding IMFs from all decomposition trials.Reconstruct the original signal as $x\;[n]=\sum_{k=1}^{K}{\mathrm{IMF}}_{k}[n]+r_{K}[n]$


**Figure 4:**
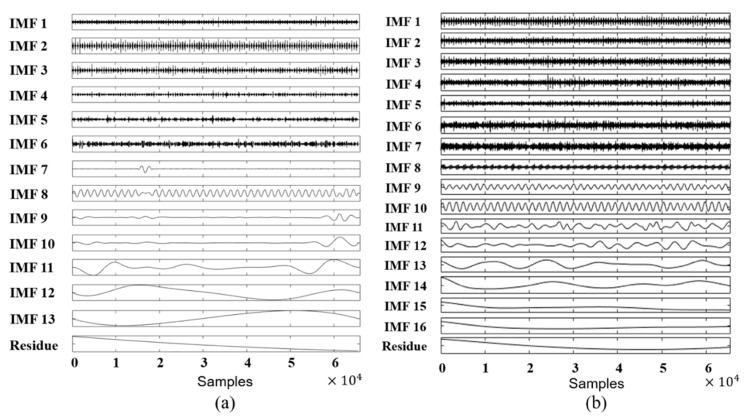
Decomposition of a one-second intensive rubbing signal by (**a**) EMD and (**b**) EEMD.

Figure 4 shows the IMFs obtained by conventional EMD and EEMD for a signal corresponding to an intensive rubbing condition. Figure 4a shows that IMF 8, which corresponds to the fundamental frequency, contained an additional oscillatory component that affects the quality of information carried by this mode, an example of mode-mixing. In this figure, IMF 9 and IMF 10 correspond to the 1/2× and 1/3× fractional harmonics; however, the behavior of these oscillations looks very similar. Figure 4b demonstrates that EEMD better separated the extracted modes, and the IMF of the fundamental frequency (IMF 10) is not affected by other oscillations or noise. Also, EEMD was able to decompose the original vibration signal more accurately into a larger number of modes. IMF 9, the 3/2× fractional frequency harmonic, became visible after decomposition, while in conventional EMD decomposition the component with this frequency harmonic was absent. Moreover, better separation of the IMFs was observed in the range of low fractional harmonics, such as 1/2× and 1/3×. These results are reasonable because due to its specific features as an improvement to the traditional EMD approach, EEMD is able to better separate oscillating components and deliver more clear IMFs.

### 2.3. IMF Selection Procedure for Rubbing Fault Diagnosis

As discussed in Section 1, the extracted IMFs can be either signal-dominant or noise-dominant, so it is crucial to select informative IMFs that contain intrinsic information about rub-impact faults. This paper presents a novel adaptive selection method for informative signal-dominant IMFs that can be applied to the domain of rubbing signals. The following two procedures must be applied in IMF selection: evaluating the extracted components using certain criteria to determine which candidate IMFs contain the most valuable information and creating a subset that contains those chosen IMFs. To define the evaluation criterion, we carefully analyze the rubbing phenomenon using the DPR of rub-impact and the KLD (a statistical similarity metric) of each extracted IMF. Based on this new criterion, all of the components can be sorted by their relevance to the rubbing process. Once the IMFs are sorted and their quality determined, the best adaptively selected candidates are used to reconstruct a signal with reduced noise that contains clear rub-impact fault components and can be used for rubbing fault feature extraction.

Since it is known that the appearance and behavior of fractional harmonics are essential features for rub-impact fault diagnosis [11,12,38], the better extraction and separation of the modes containing these harmonics make EEMD preferable to EMD.

**Figure 5:**
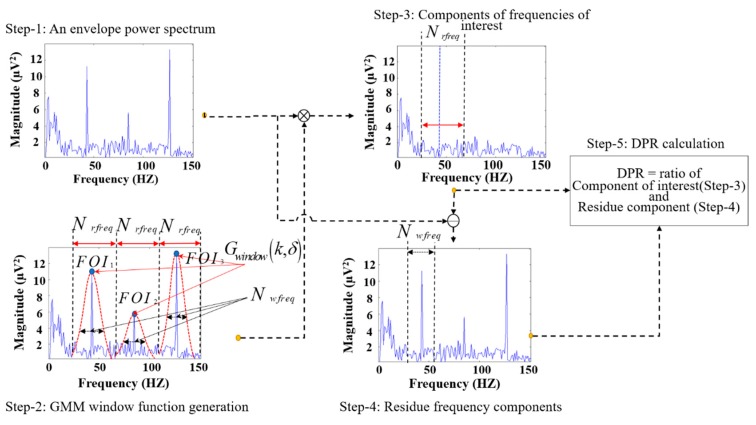
Evaluation of the IMFs by computing the degree-of-presence ratio (DPR) over the frequency of the interest and its harmonics. GMM, Gaussian mixture model; FOI, frequency of interest.

Whenever rub-impact faults occur in a system, they affect the harmonics of the fundamental frequency and some fractional frequencies, which can be clearly observed in the envelope power spectrum of the signal. DPR aims to detect the presence and power of these expected frequencies and their harmonics in the envelope power spectra of the IMFs and quantifies each IMF with respect to the ratio of the presence using the procedure presented in Figure 5. To ensure that the DPR can determine informative and meaningful IMF components, the important frequencies of interest and their harmonics were determined based on various studies of rubbing processes [11,12,38] to be the following: 1/3×, 1/2×, 2/3×, 1×, 4/3×, 3/2×, and 5/3×. The detailed DPR calculation procedure is as follows.

**Step 1:** To compute an envelope power spectrum, the analytical signal of the IMF in the time domain is first calculated. For example, if x(t) is the original IMF signal, the analytical signal can be represented as a combination of the original IMF signal and the virtue of the Hilbert transform. The analytical signal can thus be formulated as follows:

(2) $$x^{h}\left(t\right)=x\left(t\right)+i\;\tilde{x}\left(t\right),$$

where $\tilde{x}\left(t\right)$ is the Hilbert transform. Convolution of the original IMF signal with the signal 1/πt yields the following:

(3) $$\tilde{x}\left(t\right)=x\left(t\right)*\frac{1}{2\pi }=\frac{1}{\pi }\int_{-\infty }^{\infty }h\left(t\right)\frac{dt}{t-\tau }.$$

The power spectrum of the analytical signal is then obtained by taking the square of the absolute value of the Fourier transform, ${\left|F\left\{x^{h}\left(t\right)\right\}\right|}^{2}$, as shown in Step 1 of Figure 5.

**Step 2:** Since the envelope power spectrum reveals the transient impact of rubbing, a Gaussian mixture model (GMM) window $\left(G_{window}\left(k,\delta \right)\right)$ is constructed around the peaks of the frequencies of interest and their integer multiples to attain residual components in the frequency domain of the envelope power spectrum. The coefficients of $G_{window}\left(k,\delta \right)$ are computed as follows:

(4) $$\begin{array}{l} G_{window}\left(k,\delta \right)=\sum_{i=1}^{n}\exp\left(-\frac{1}{2}\;\left(\delta \frac{{\left(k-{\mathrm{FOI}}_{n}\right)}^{2}}{\left(N_{rfreq}/2\right)}\right)\;\right)\\ s.t.\;{\mathrm{FOI}}_{n}-f_{range}\leq k\leq {\mathrm{FOI}}_{n}+f_{range}. \end{array},$$

where ${\mathrm{FOI}}_{n}$ defines the $n_{th}$ harmonic of the frequency of interest; n=3 was used to calculate the DPR. $N_{rfreq}$ is the number of frequency bins in the range ${\mathrm{FOI}}_{n}-f_{range}\leq k\leq {\mathrm{FOI}}_{n}+f_{range}$, as shown below:

(5) $$N_{rfreq}=2.f_{range}/f_{resolution}\;.$$

The value of $N_{wfreq}$ can be computed (see Step 2 in Figure 5) for a GMM-DPR as follows:

(6) $$\begin{array}{l} N_{wfreq}=\left(\left(2\times 2/100\right)\times \mathrm{FOI}\right)/f_{resolution},\\ s.t.\;{\mathrm{span}}_{FOI}=\left(2/100\right)\times \mathrm{FOI}. \end{array}.$$

Similarly, δ is a Gaussian random variable that is inversely proportional to the standard deviation and can be defined as follows:

(7) $$\delta =\left(N_{rfreq}/N_{wfreq}\right)\sqrt{2\ln\;m}.$$

Here, $N_{wfreq}$ is the frequency bin size around the frequency-of-interest components, and the value of m is constant in the range 0<m<1, (m=0.1). A narrow band frequency range (i.e., $f_{range}=1/3\mathrm{FOI}$) is used for all the frequencies of interest in this paper.

**Step 3**: The components of the frequencies of interest are then calculated by multiplying the defined Gaussian window $G_{window}\left(k,\delta \right)$ around the frequencies of interest and their harmonics in the attained envelope power spectrum.

**Step 4:** The residual frequency components are computed by subtracting the frequency-of-interest component (from Step 3) from the envelope power spectrum. Once we have the frequency of interest and residual components, the DPR is calculated as the ratio of the frequency-of-interest components and the residual frequency components as shown below:

(8) $$\mathrm{DPR}=10\cdot \log\left(\sum_{n=1}^{3}\left\{\sum_{j=1}^{N_{wfreq}}C_{n,j}^{2}/\sum_{j=1}^{N_{rfreq}}R_{n,j}^{2}\right\}+10\right)\left(dB\right).$$

Here, $C_{n,j}$ and $R_{n,j}$ are the magnitudes of the $j_{th}$ frequency bin for the frequency-of-interest components and residual frequency components, respectively, around the $n_{th}$ harmonic of the frequency of interest.

Note that a large DPR of an IMF means that the component contains valuable information about the behavior of the fundamental frequency, its fractional harmonics, and other frequencies that can be observed during the rubbing process. A small DPR value indicates the opposite: the current IMF could be noise-dominant and does not provide valuable information that can be used for rubbing fault diagnosis.

Next, the distances between the probability density functions (PDFs) of the extracted IMFs and the original signal are calculated to discover similarities between the hidden structures and determine how much of the original signal’s information is preserved in the selected component.

The distance function utilized in this paper, KLD, is one of the most frequently used information-based distance measures and is a member of the Shannon’s entropy family [39]. The function is defined as follows:

(9) $${\mathrm{KLD}}_{k}=\sum_{x\in X}\mathrm{PDF}(x(t))\;ln\frac{\mathrm{PDF}(x(t))}{{\mathrm{PDF}(\mathrm{IMF}}_{k}(t))},$$

where PDF(x(t)) is the PDF of the original signal x(t) and $\mathrm{PDF}\left({\mathrm{IMF}}_{k}\left(t\right)\right)$ is the PDF of the $k^{th}$ extracted IMF.

Once the DPR and KLD have been calculated, we define an IMF quality measure by Equation (10), which will be used to determine the relevance of the extracted components, and then select informative modes:

(10) $$\mathrm{IMF}val_{k}={\mathrm{DPR}}_{k}/{\mathrm{KLD}}_{k}.$$

Here, ${\mathrm{DPR}}_{k}$ represents the sum of all degrees of presence calculated for each $N_{wfreq}$ and their three harmonics for the $k^{th}$ IMF, and ${\mathrm{KLD}}_{k}$ is the measure of how close the PDF of the $k^{th}$ IMF is to the PDF of the original signal.

Finally, the IMF selection procedure is completed using a thresholding technique that compares the ratio $\mathrm{IMF}val_{k}$ with a threshold value that is set to 1. The threshold value is assigned to 1 because the relationship between the DPR and KLD for each IMF can be considered as a signal-to-noise ratio (SNR). It is known that when the SNR is greater than 1, the amount of signal in the data is greater than the amount of noise. That is, a small KLD distance value in the $\mathrm{IMF}val_{k}$ ratio indicates that the PDF of the mode under evaluation is similar or close to that of the original signal. Therefore, $\mathrm{IMF}val_{k}$ grows when the DPR (the metric that reflects the presence of important information) is large compared to KLD. This means that the IMF contains a significant amount of valuable information about the rubbing fault and its PDF is very close to that of the original signal, which indicates that the IMF is signal-dominant. In the opposite direction, $\mathrm{IMF}val_{k}$ decreases when the IMF contains a small amount of valuable information (low values of DPR) and a large KLD (the component appears to be noise-dominant). Therefore, if $\mathrm{IMF}val_{k}$ is greater than or equal to 1, the IMF will be included in the optimal subset for signal reconstruction; otherwise, it will not be used. Using this proposed IMF quality measure and threshold-based selection approach, all of the extracted components can be evaluated and the most valuable modes are adaptively selected based on their relevance to rubbing processes and the amount of information presumed from the original signal.

### 2.4. Feature Extraction and Configuration of Feature Set

The proposed IMF selection process provides a set of the most meaningful IMFs that carry important information about the rubbing processes present in the original signal. We then utilize these selected IMFs for signal reconstruction (i.e., obtaining a noiseless rubbing signal) and feature extraction. The reconstructed signal can be obtained using the following equation:

(11) $$x_{rec}(t)=\sum_{l=1}^{N}\mathrm{IMF}.$$

Here, N is the total number of selected IMFs and l corresponds to the order number of each selected mode.

After signal reconstruction, time-domain statistical features in the reconstructed signal and frequency-domain features in its complex envelope power spectrum are extracted. These features are considered to be discriminative because significant changes in the amplitude of the fundamental frequency and its fractional harmonics are usually observed in envelope power spectrum when a rub-impact fault occurs. The amplitude of the vibration signal in the time-domain also changes due to the increased fluctuations of the signal wave depending on the rubbing fault intensity. Thus, the changes in signal behavior and its energy with variations in rubbing intensity levels can be well-characterized by extracting dimensional time-domain statistical feature parameters, such as the root mean square (RMS), kurtosis, skewness, and square root of the amplitude (SRA) from the signal reconstructed using the sets of selected IMFs. These features are widely used as health indicators of various systems in fault diagnosis problems and can provide a good insight into rubbing processes in the time domain because they are known to be features sensitive to impulse faults [40]. Here, skewness and kurtosis are the third and fourth central moments of standard deviation. These two features are known as statistical indicators sensitive to the degree of peakedness of the signal that can be used well to characterize the variability of the vibration signal in the time domain when a rubbing fault occurs in a system. Also, the RMS and SRA are used in this study to describe the changes in intensity levels of the vibration signal affected by a rub-impact fault. Note that the RMS and SRA are usually both thought of as statistical parameters that reflect the behavior of the signal’s amplitude in different scales, so these two features may not provide drastically different information, but they complement each other when used simultaneously.

Additionally, frequency domain analysis can help to discover some information that cannot be observed in the time domain [41]. Features that involve statistical properties of frequency are extracted from the envelope power spectra of the reconstructed signals. As we know, the frequency spectra of the rub-impact signals obtained by conventional frequency-domain signal analysis techniques, such as FFT, fail to present relevant information about rubbing processes. Various studies [20,23] have shown that the frequency spectra of the original rub-impact signals obtained by FFT usually cannot represent the frequency harmonics relevant to rub-impact faults, although the fundamental frequency and some high-order harmonics still may be present. The frequency-domain features extracted in this way cannot be considered as good features for diagnosing rub-impact faults because the behavior of the fundamental frequency and its higher harmonics can be affected by various mechanical faults in rotational machinery as well as properties of the environment in which the machine is installed. It is therefore difficult to verify whether these extracted features are actually related to rubbing faults or not. On the other hand, the frequency-domain features extracted in this study after time-frequency EEMD decomposition of the original vibration signal followed by informative IMF selection are definitely capable of reflecting rub-impact faults because the fault information is highly detectable and well-presented by the envelope spectra of the signals reconstructed using sets of selected IMFs. Moreover, it was observed that the valuable rubbing fault-related harmonics are detectable and well-localized in the envelope power spectrum of the reconstructed signal. Thus, the frequency-domain features extracted in this study are as follows: mean frequency, frequency RMS, and frequency standard deviation (St. Dev). Here, the mean frequency value corresponds to the mean of the frequency of the envelope power spectrum computed for a reconstructed signal, frequency RMS characterizes the intensity of the signal in the frequency domain, and frequency St. Dev describes the deviation of the signal from its main frequency components in the frequency domain.

Due to the extraction of the feature parameters from both time and frequency domains, the proposed feature model can be considered as a hybrid one. The basic idea beyond this model is that each feature extracted from both domains aims to characterize and quantify the specific physical and statistical properties of the denoised vibration signal affected by rub-impact. Furthermore, the features used in this study are well-recognized and frequently used health indicators in other problems of vibration-based condition monitoring, such as rolling-element bearing fault diagnosis [42] and prognosis [43]. Concretely, frequency-domain features, such as mean frequency, frequency RMS, and frequency standard deviation, are frequently applied for rolling element bearings (REB) fault diagnosis, whereas the time-domain RMS, kurtosis, and skewness are popular health indicators used for fault prognosis and remaining useful lifetime estimation. All the extracted features are shown in Table 2.

## 3. Experimental Results and Discussion

The effectiveness of the proposed rub-impact fault feature extraction technique with its novel IMF selection procedure is presented along with its possible application to fault diagnosis.

### 3.1. Training and Testing Data Configuration

An appropriate training and testing dataset configuration is important in order to determine the generalized quality of the proposed fault diagnosis approach. In this paper, a rub-impact fault was simulated using a shaft imbalance produced by adding extra weights to the NDE. In total, 10 different weights (i.e., rubbing intensities) were added to the shaft, namely 0, 0.5, 1.0, 1.5, 1.6, 1.7, 1.8, 2.0, 2.4, and 2.8 g. A 59-s-long signal was acquired for each intensity level. Therefore, the created dataset contained 590 signal instances in total. In this paper, the set of features extracted for the experimental part consisted of a total of $N_{c}\times N_{s}\times N_{f}$ features. Here, $N_{c}$ is the number of bladed rotor intensity classes (conditions) simulated in this study, $N_{s}$ is the number of instances for each condition, and $N_{f}$ is the number of extracted features.

In this paper, the k-fold cross-validation (k=3) procedure was applied for validating the introduced methodology during each experimental trial. In the k-fold cross-validation method, the entire dataset is randomly split into k-folds, where each fold should be used once as a testing subset applied to a classifier trained on the remaining k − 1 subsets. Specifically, in this paper, 39 randomly chosen data samples from each class were selected as the training subset and the remaining 20 data instances from each class were used to construct the testing subset. Thus, each training subset contained 390 instances and each testing subset consisted of the 200 remaining data samples.

### 3.2. Validation of the Selected IMFs Using the Proposed Approach

The proposed IMF selection method allows for derivation of the specific objective function values that can be used to evaluate the quality of the components extracted by EEMD; these values are presented in Table 3. As the result of the introduced procedure, the sets containing the most valuable components were obtained for each signal class as shown in Table 4. In this study, rubbing faults were caused by imbalance in the testbed shafts, where the imbalance was created by attaching extra weight to the NDE. As shown in Figure 3 in Section 2.1, the increase in weight results in an increased degree of shaft imbalance, which causes the various rubbing conditions observed during the experiment. It was also observed that some rubbing conditions, which were validated using a thermal camera, had different degrees of imbalance due to the additional weight. This observation explains why our IMF selection method adaptively delivered subsets with various components for each class; however, each subset was also shown to contain IMFs that were common for all classes.

**Figure 6:**
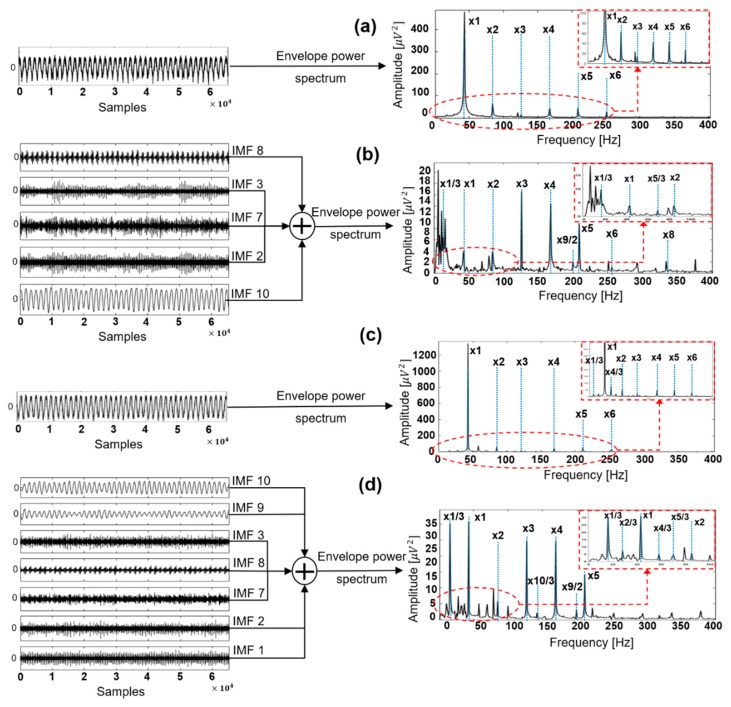
Envelope power spectra of (**a**) class ‘0 g’ original signal; (**b**) reconstructed signal using selected IMFs for class ‘0 g’; (**c**) class ‘2.8 g’ original signal; and (**d**) reconstructed signal using selected IMFs for class ‘2.8 g’.

Since the modes obtained by the decomposition are amplitude-modulated, the envelope power spectrum of the reconstructed signal must be computed to verify the quality of the subsets provided by IMF selection. Figure 6 demonstrates the envelope power spectra of the original and reconstructed signals using the subsets of selected IMF components corresponding to the intensity classes produced by 0 g and 2.8 g of extra weight. As shown in Figure 6a, it was observed that the envelope power spectrum of the original signal when no rubbing occurred mostly consisted of the fundamental frequency and its high-order harmonics. No fractional harmonics were clearly observed in the power spectrum. Also, the peak amplitude of the fundamental frequency harmonic was drastically higher than the peak amplitudes of other harmonics present in the power spectrum. Figure 6b shows the envelope power spectrum of the reconstructed signal when no rubbing occurred in the system. This power spectrum includes the fundamental frequency 1× and its high-order harmonics, while the majority of fractional harmonics are not present or their amplitudes are very small, which is reasonable for a system in this state. The peak amplitudes of the harmonics present were also relatively small. Figure 6c presents the envelope power spectrum of the original signal acquired during a severe rub-impact fault. From this figure it is seen that the main frequency 1× and its multiple harmonics were clearly present in the envelope power spectrum. Moreover, some of the fractional harmonics that are considered evidence of the rubbing process, 1/3× and 4/3×, were barely seen in this figure. Even though these fractional harmonics were present, the problem with extracting features for rubbing fault diagnosis from this kind of power spectrum is that the amplitude of the fundamental frequency is noticeably higher than the amplitudes of all other harmonics. Therefore, the numerical values of features extracted from this original signal mostly reflect the behavior of the main frequency, while the influence of the fractional frequencies will be negligible. It is known that many various mechanical faults in rotating machinery affect the behavior of the fundamental frequency. Therefore, when the only signs of a fault are amplitude changes of the fundamental frequency and its high-order harmonics, it is not easy to determine whether the extracted features actually reflect the rubbing process or whether they are related to other mechanical faults or the environmental features. In contrast, Figure 6d shows that the frequencies which appeared in the envelope power spectrum of the reconstructed signal of the severe rub-impact fault contained important information corresponding to the various signal harmonics, such as the fundamental frequency 1×, its high-order harmonics 2×, 3×, 4×, and 5×, and the fractional harmonics 1/3×, 2/3×, 4/3×, 5/3×, 10/3×, and 9/2×, which are considered valuable features for rub-impact faults [11,12,38]. Furthermore, the amplitudes of the fundamental frequency component and its high-order harmonics during the severe rubbing process were higher than those shown in Figure 6b. These observations reveal that the selected IMF subsets contain meaningful information and that the features extracted from the reconstructed signals well-represent rubbing faults of various intensities.

**Figure 7:**
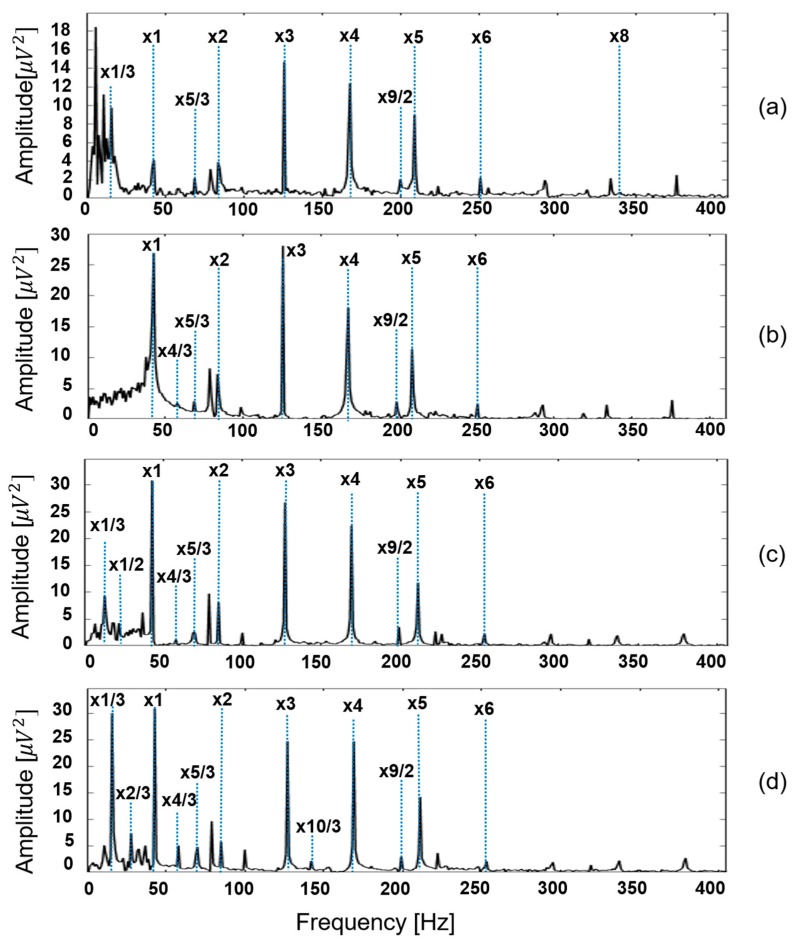
Envelope power spectra of reconstructed signals obtained using EEMD and the proposed IMF selection technique for classes (**a**) ‘0.0 g’; (**b**) ‘1.6 g’; (**c**) ‘2.0 g’; and (**d**) ‘2.8 g’.

**Figure 8:**
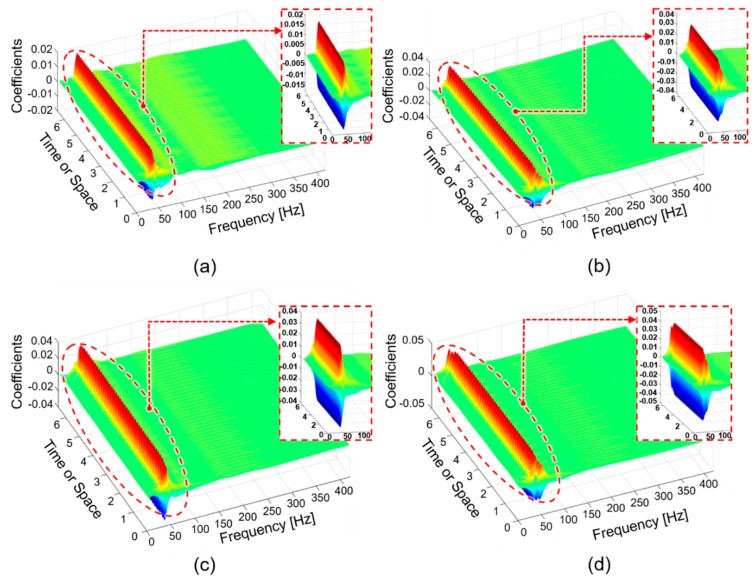
Fast Fourier Transform (FFT) spectra and wavelet maps of signals corresponding to classes (**a**) ‘0.0 g’; (**b**) ’1.6 g’; (**c**) ’2.0 g’; and (**d**) ‘2.8 g’.

To validate the efficacy of EEMD decomposition followed by informative IMF selection in representing rub-impact faults, we compared the envelope power spectra of the signals reconstructed using the selected IMFs with wavelet maps obtained by applying a continuous wavelet transform (CWT) to the original signals as described in [44]. Figure 7 shows the envelope power spectra obtained after signal reconstruction for rubbing intensities corresponding to classes ‘0 g’, ’1.6 g’, ’2.0 g’, and ‘2.8 g’. The wavelet maps corresponding to the same signals obtained using CWT are shown in Figure 8.

Comparing Figure 7 and Figure 8, it is clear that the envelope power spectra of signals reconstructed using the selected IMFs after EEMD decomposition provide better information about rubbing faults than the wavelet maps obtained by CWT. Specifically, from Figure 7 it can be seen that the appearance of fractional harmonics and their amplitude behavior, combined with information about the fundamental frequency and its high-order harmonics, allows for the differentiation of rub-impact faults of various intensities as well as differentiation of the normal state of the system with no rubbing faults. Figure 8 shows that it while might be possible to discriminate between the state of the system when a rubbing fault is and is not present in a signal, it might be difficult to differentiate between rubbing faults of various intensities. It is clear that the most significant difference between the wavelet map patterns and the wavelet coefficient scales for the various states is observed between the case where there is no rub-impact fault in a system (Figure 8a) and that in which severe rubbing is present (Figure 8d). The wavelet map patterns and the scales of the wavelet coefficients for classes corresponding to slight rubbing and intensive rubbing were very similar. Moreover, from the wavelet maps it is difficult to verify whether the frequency harmonics inherent to rub-impact faults are present in the FFT spectrum due to significant overlap of the harmonics around the fundamental frequency. Therefore, it can be concluded that the method proposed in this paper is capable of representing and delivering valuable information about rub-impact faults of various intensities better than conventional CWT.

### 3.3. Performance Evaluation of the Proposed Rubbing Fault Feature Extraction Scheme with the New IMF Selection Procedure

To evaluate the quality of the features extracted from the reconstructed signal and their ability to provide sufficient information about rub-impact faults with different intensity levels in rotor blades, we compared our feature extraction approach with three TFA feature extraction methods that use numerical-valued features for rub-impact fault diagnosis. The first TFA method utilizes wavelet packet transform and maximum SV computation to extract features from signals containing rubbing faults (referred to as WPT + MSV) [4]. The second TFA combines conventional EMD decomposition and maximum SV extraction to create a set of features for differentiating rubbing faults (referred to as EMD + MSV) [24]. The third TFA approach applies digital wavelet transform (DWT) to the rubbing fault signal and then uses decomposed sample data in the time domain of the third level of transformation as feature vectors for diagnosing rub-impact faults (referred to as DWT + TDSIG) [5]. Also, for comparison purposes, we applied our proposed hybrid feature model to signals reconstructed using sensitive IMFs selected by the method presented in [30] (referred to as SensIMF + HFM). In this paper, the one-against-all multiclass SVM (OAA-MCSVM) classifier [35,36] was employed to perform the comparison of the above approaches. To ensure the repeatability of the results and exclude the effect of randomness, our experiments were performed 20 times with different combinations of training and testing data.

The classification accuracy for each group of samples was computed using the true positive rate evaluation index (TPR), which is given below:

(12) $${\mathrm{TPR}}_{l}=\left(1/k\right)\sum_{i=1}^{k}\left(N_{TP}^{i,l}/\left(N_{TP}^{i,l}+N_{FN}^{i,l}\right)\right)\times 100\;(\%),$$

where k is the total number of cross-validation folds (k=3), $N_{TP}^{i,l}$ is the total number of samples in class l that are correctly classified as class l, $N_{FN}^{i,l}$ is the number of samples within class l that are not recognized as class l, and i indicates the i-th iteration of the k-fold cross-validation procedure. The final TPR for each class presented here is computed as an average of the TPRs achieved over 20 experiments. The standard deviation (St.Dev) of the TPR was also calculated and shown in the results.

The classification accuracy (CA) for each experiment was determined using the relation given below:

(13) $$\mathrm{CA}=\left(1/k\right)\sum_{i=1}^{k}\left(\left(\sum_{l=1}^{M}N_{TP}^{i,l}\right)/N_{samples}\right)\times 100\;(\%),$$

where M is the total number of groups and $N_{samples}$ is the total number of samples represented in a particular testing subset. The final classification accuracy presented in the results is the average classification accuracy (ACA) achieved over 20 experiments. The standard deviations (St.Dev) of the CA metric were also calculated and presented in the results.

The experimental results are shown in Table 5. These results demonstrate that rub-impact fault feature extraction using EEMD with adaptive IMF selection based on the novel presented mode evaluation metric for rubbing faults and the proposed hybrid feature model outperforms the reference methods in terms of average classification accuracy, with an accuracy of 99.8% over 20 experiments. Table 5 shows that the average TPR values of the proposed method over 20 experiments were over 99.5% and the standard deviations of the TPR did not exceed 1.5%.

**Figure 9:**
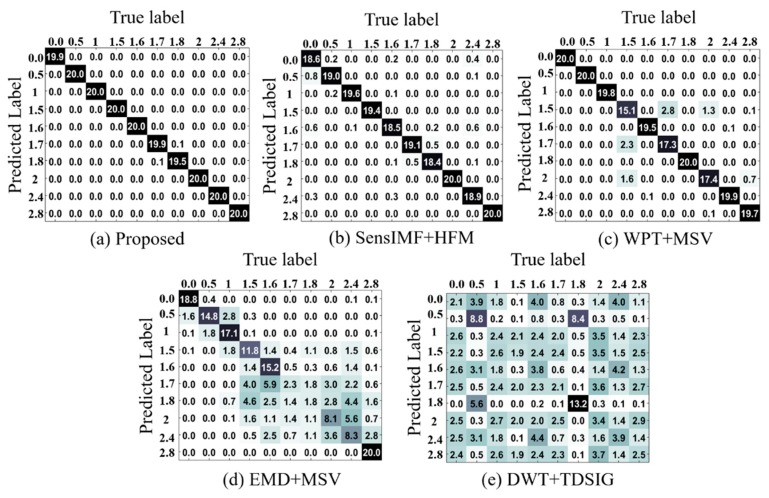
Confusion matrices for classification using (**a**) proposed; (**b**) SensIMF + HFM [30]; (**c**) WPT + MSV [4]; (**d**) EMD + MSV [24]; and (**e**) DWT + TDSIG [5] approaches. All results are presented as the average of 20 experiments.

Confusion matrices for the proposed framework and the reference methods are presented in Figure 9. The confusion matrix is a robust technique that provides a visualization of the performance of a classifier algorithm in terms of the deviation between actual and predicted results [35]. According to the results in Figure 9a, the proposed method perfectly identified rubbing faults of all intensities with a very low misclassification rate compared to its counterparts: SensIMF + HFM [30] in Figure 9b, WPT + MSV [4] in Figure 9c, EMD + MSV [24] in Figure 9d, and WT + TDSIG [5] in Figure 9e.

These results can be explained as follows. EEMD decomposition is capable of extracting clear ‘true’ IMFs, which can be easily associated with frequency bands of the original signal. Importantly, the proposed adaptive IMF selection method for rubbing signals, in conjunction with the novel IMF evaluation technique, enables meaningful intrinsic components to be precisely determined. These components include valuable information about the frequency bandwidths containing specific frequency peaks and their harmonics, which are considered to be evidence of rubbing processes in a signal [11,12,38].

The reference feature extraction approaches for rub-impact fault diagnosis resulted in average classification accuracies of 96.6%, 95.0%, 60.0%, and 22.6%, respectively.

Regarding SensIMF + HFM, Table 5 shows that the average classification accuracy achieved by this method for classifying rubbing faults of various intensities was slightly less than one, made by the proposed methodology. Note that SensIMF + HFM is a synthetic method that combines a sensitive IMF selection method for rub-impact fault diagnosis [30] and our proposed hybrid feature model extracted from signals reconstructed using the selected sensitive IMFs. The results presented in Table 5 and Figure 9b demonstrate that this combined approach performs well at differentiating various rubbing conditions, but is still slightly worse than the method proposed in this paper. The difference between the results of the two methods can be explained as follows. Although both IMF selection methods were introduced for rub-impact fault diagnosis, the main concepts behind them are different. The method proposed in this paper aims to select the IMFs that are directly related to rubbing faults in the system by both searching for the specific rub-impact fault harmonics in the envelope power spectra of IMF candidates and computing the KLD similarity between the extracted components and the original non-decomposed signal. On the other hand, the referenced method relies on computing ‘sensitivity factors’ of IMFs using only the correlation between the extracted components and the original signal as well as the signal acquired under the normal operating conditions of rotating machinery [30]. Considering that both methods utilize the same feature model, it can be concluded that the approach proposed in this paper is able to better highlight the important frequency harmonics of rubbing faults than a method that relies only on correlation properties of signals and IMFs without considering the exact information content carried by those modes.

Regarding WPT + MSV, the average accuracy was smaller than that of the proposed feature extraction approach. Table 5 and Figure 9c show that in general, the WPT + MSV approach deals with differentiating rubbing faults of various intensities well; however, for the rubbing intensities corresponding to extra weights of 1.5 g, 1.7 g, and 2.0 g attached to the NDE, the TPR values were relatively low with increased standard deviation. These results can be explained because as mentioned above, rubbing faults in this study were simulated by adding extra weight to the NDE. This means that with increasing rotor imbalance, the rubbing becomes more severe and the characteristics of the vibration signal change significantly. The quality of the wavelet packet transform strongly depends on the choice of mother wavelet function. Moreover, since this approach is not entirely adaptive and data-driven, the same mother wavelet function simply cannot perfectly match all of the changing types of vibration signals which contain both rotor imbalance and a complex nonlinear fault, such as a rub-impact fault.

Regarding EMD + MSV, Table 5 and Figure 9d show that the average accuracy and TPR achieved by this method over 20 experiments were low compared to the proposed method, SensIMF + HFM, and WPT + MSV. There might be two main reasons for this. The first is the problem of mode-mixing which is inherent to the conventional EMD approach. A rub-impact fault is known to be a complex fault that induces various nonlinear processes in the signal, and due to the mode-mixing problem, the quality of the extracted modes may not be good enough to extract discriminative features. In the ideal case, each extracted component should represent a specific frequency band so that the extracted features can describe the processes in the system well. However, mode-mixing causes the mixing of harmonics in the frequency spectra of the obtained IMFs. This makes it difficult to clearly understand the physical meaning of each mode, and the quality of the extracted features becomes poor. The second reason for the obtained results is that the EMD + MSV feature extraction approach does not consider the selection of valuable IMFs. The cardinality of extracted IMFs is usually large, and they can be either signal- or noise-dominant. In the case of diagnosing rub-impact faults, it is important to determine which of the extracted components can provide useful and clear information because the features extracted from noise-dominant components can expand the feature models and degrade the overall classification performance by delivering poor-quality or redundant features that are not related to the fault being investigated.

In addition, in both WPT + MSV and EMD + MSV it might be difficult to verify whether the extracted SVs as features actually reflect rub-impact faults or whether their values are more closely related to the properties and setup of the system because these environment properties are also reflected in the acquired signal.

Regarding DWT + TDSIG, its performance for differentiating rub-impact faults of various intensities was not satisfactory in this study. Table 5 shows that this method achieved an average classification accuracy of 22.6% over 20 experiments. Also, the TPR values were noticeably lower and the standard deviations were significantly higher than those demonstrated by the other methods presented. The confusion matrix in Figure 9e shows a similar result. Such a poor performance can be explained as follows. First of all, choosing a wavelet function is not an easy task but is important because the quality of wavelet-based decomposition is directly affected by the chosen mother wavelet. Furthermore, the dataset used in this study contains two mechanical faults: a rub-impact fault and its cause, an imbalance of the shaft. The wavelet function chosen in the referenced method may not match all the signal groups present in the dataset. This method also does not propose any technique for adaptive selection of a proper wavelet level band that contains the most essential information about the mechanical fault after decomposition. Finally, the poor performance seems to be caused by the use of time-domain decomposed sample data as a feature vector to the OAA-MCSVM classifier. Although SVM is known to be a robust classifier that is insensitive to the dimensionality of the feature vector [45], the use of a time-domain signal instead of statistical feature parameters creates a feature vector of huge dimensions. This leads the classifier to attempt to create a very complex hyperplane to try to separate the signal samples, causing the final classification accuracy to be poor. Of course, DWT incorporates some downsampling while decomposing the original signal, but this has benefits only when the original signal is relatively short (e.g., 2048 sampling points [5]). If the original signal is long like those used in this study, even after the downsampling performed by DWT, the remaining signal at the third level of decomposition is still long.

Overall, the proposed feature extraction methodology is highly useful for the diagnosis of rub-impact faults of various intensities because of its main concept: informative IMF selection with a novel IMF quality metric and a hybrid feature extraction procedure based on a signal reconstructed using the selected IMFs in which fault symptoms are highly observable.

## 4. Conclusions

This paper presented a new feature extraction method for rub-impact fault diagnosis based on EEMD with an informative IMF selection procedure and hybrid feature model. The proposed method addresses the problems of both efficient feature extraction and the selection of meaningful and proper IMFs suitable for rub-impact fault diagnosis. First, EEMD provided well-separated and well-behaved oscillating components of the original vibration signal and reduced the influence of the mode-mixing that is inherent to the conventional EMD approach. Second, subsets of meaningful signal-dominant modes were selected by the proposed IMF selection method using a novel quality measure based on the DPR and the Kullback–Leibler divergence distance metric. Finally, the adaptively selected IMFs were used to reconstruct a noiseless and clear rubbing fault signal for hybrid feature extraction and construct the feature set. In the experimental part of this study, the proposed set of features was used to differentiate rub-impact faults with various intensities using the OAA-MCSVM classifier. The experimental results demonstrated that the proposed combination of signal decomposition and our IMF selection technique is capable of extracting relevant information about rubbing faults of various intensities. The proposed feature extraction approach outperformed reference methods in terms of classification accuracy and TPR. Fault classification using the features extracted by the proposed method achieved an average accuracy of 99.8% over 20 experiments with various combinations of training and testing data. In our future research work, we would like to elaborate more on the thresholding technique used in the proposed IMF selection approach. Specifically, the use of adaptive thresholding may reduce the number of selected IMF components for various classes, and hence, possibly lead to more ‘clean’ reconstructed signal. Also, the evaluation of the proposed methodology in other fault detection and diagnosis fields is the scope of future work.

## Figures and Tables

**Table 1:** Data acquisition (DAQ) system specifications.

Displacement Sensors (3300 XL NSv)	**Frequency range:** 0 to 10 kHz **Sensitivity:** 7.87 V/mm (200 mV/mil) +12.5%/−20%
DAQ System (Pulse 3560 C)	**Generator:** Input/Output 4/2 ch. module Input/Output 5/1-ch. controller module **Frequency range:** 0 to 25.6 kHz

**Table 2:** Time- and frequency-domain statistical feature parameters. Where $x_{rec}$ is the reconstructed signal in the time domain, f is the spectral component of the reconstructed signal $x_{rec}$, and σ is the standard deviation of the reconstructed signal $x_{rec}$. RMS, root mean square.

Parameters	Equations	Parameters	Equations
Root mean square $\left(f_{1}\right)$	$\sqrt{\left(\frac{1}{N}\right)\sum_{i=1}^{N}x_{rec}{}^{2}(i)}$	Square root of the amplitude $\left(f_{2}\right)$	${\left(\left(\frac{1}{N}\right)\sum_{i=1}^{N}\sqrt{\left|x_{rec}\right|}\right)}^{2}$
Skewness $\left(f_{3}\right)$	$\left(\frac{1}{N}\right)\sum_{i=1}^{N}{\left(\frac{\left(x_{rec}(i)-\bar{x_{rec}}\right)}{\sigma }\right)}^{3}$	Kurtosis $\left(f_{4}\right)$	$\left(\frac{1}{N}\right)\sum_{i=1}^{N}{\left(\frac{\left(x_{rec}(i)-\bar{x_{rec}}\right)}{\sigma }\right)}^{4}$
Mean frequency $\left(f_{5}\right)$	$\left(\frac{1}{N}\right)\sum_{i=1}^{N}f(i)$	RMS frequency $\left(f_{6}\right)$	$\sqrt{\left(\frac{1}{N}\right)\sum_{i=1}^{N}f_{}^{2}(i)}$
Frequency standard deviation $\left(f_{7}\right)$	$\sqrt{\left(\frac{1}{N}\right)\sum_{i=1}^{N}{\left(f_{}(i)-f_{5}\right)}^{2}}$		

**Table 3:** The objective values of the proposed IMF selection metric calculated for all the extracted components for each signal class. The values highlighted with bold font correspond to the selected valuable IMFs.

$\;IMFval_{k}$											
	Class	0.0	0.5	1.0	1.5	1.6	1.7	1.8	2.0	2.4	2.8
k											
**1**		0.12	0.23	0.92	**1.2**	0.75	**1.44**	**1.47**	**2.24**	0.78	**1.64**
**2**		**1.66**	0.77	**2.47**	**1.8**	**1.12**	**3.57**	**3.9**	**2.85**	**2.2**	**2.98**
**3**		**2.3**	**3.86**	**20.6**	**19.2**	**2.52**	**21.9**	**7.24**	**28.3**	**2.44**	**20.4**
**4**		0.17	0.09	0.24	0.17	0.55	0.31	0.29	0.34	0.22	0.12
**5**		0.38	0.23	0.21	0.17	0.9	0.12	0.32	0.25	0.21	0.32
**6**		0.44	0.67	0.62	0.21	031	0.71	0.35	0.19	0.21	0.26
**7**		**1.71**	**7.48**	**5.75**	**3.39**	**1.34**	**3.79**	**6.41**	**4.46**	**1.45**	**12.2**
**8**		**2.93**	**7.37**	**25.2**	**18.7**	**2.45**	**30.5**	**8.2**	**27.1**	**2.53**	**18.9**
**9**		0.96	**12.2**	**17.5**	**34.0**	**6.37**	**25.8**	**12.6**	**51.1**	**8.12**	**29.0**
**10**		**1.21**	**5.1**	**43.1**	**40.1**	**8.45**	**45.8**	**10.3**	**88.1**	**8.01**	**70.7**
**11**		0.61	0.3	0.16	0.32	0.12	0.28	0.17	0.15	0.95	0.43
**12**		0.38	0.2	0.18	0.16	0.14	0.19	0.23	0.19	0.34	0.18
**13**		0.23	0.26	0.22	0.24	0.25	0.23	0.28	0.17	0.22	0.25
**14**		0.23	0.26	0.24	0.3	0.31	0.22	0.23	0.25	0.27	0.24
**15**		0.23	0.26	0.27	0.25	0.42	0.30	0.24	0.24	0.31	0.27
**16**		0.29	0.24	0.27	0.24	0.23	0.33	0.33	0.24	0.33	0.30
**17**		0.23	0.3	0.23	0.24	0.41	0.23	0.28	0.24	0.27	0.29
**18**		-	-	-	-	-	-	0.23	-	-	-

**Table 4:** The subsets of selected IMFs obtained using the proposed IMF selection procedure.

	Classes									
	0	0.5	1.0	1.5	1.6	1.7	1.8	2.0	2.4	2.8
**Selected IMFs**	2,3,7 8,10	3,7,8 9,10	2,3,7 8,9,10	1,2,3,7,8,9,10	2,3,7,8,9,10	1,2,3,7 8,9,10	1,2,3,78,9,10	1,2,3,7 8,9,10	2,3,7 8,9,10	1,2,3,78,9,10

**Table 5:** Experimental results. TPR, true positive rate; ACA, average classification accuracy.

Method	Average TPR (St.Dev) (%)										ACA (St.Dev) (%)
	0	0.5	1.0	1.5	1.6	1.7	1.8	2.0	2.4	2.8	
Proposed	100 (0)	100 (0)	100 (0)	100 (0)	100 (0)	99.5 (1.5)	99.75 (1.1)	100 (0)	100 (0)	100 (0)	99.8 (0.14)
SensIMF + HFM	95.9 (3.8)	95.25 (1.1)	98.25 (2.4)	100 (0)	93.0 (2.9)	97.5 (2.5)	96.6 (2.9)	100 (0)	97.5 (2.9)	100 (0)	96.6 (0.33)
WPT + MSV	100 (0)	100 (0)	100 (0)	78.6 (5.6)	99.5 (1.5)	88.2 (3.5)	100 (0)	88.6 (4.37)	99.75 (1.1)	99.75 (1.1)	95.0 (0.7)
EMD + MSV	99.7 (3.3)	76.1 (7.7)	89.1 (3.5)	60.1 (9.8)	78.4 (4.9)	11.3 (4.9)	8.8 (4.0)	41.2 (10.6)	42.7 (13.0)	100 (0)	60.0 (2.13)
DWT + TDSIG	10.7 (14.3)	44.8 (17.0)	12.3 (15.1)	9.44 (15.4)	19.3 (19.1)	10.6 (14.5)	67.2 (12.8)	17.4 (19.5)	19.7 (21.2)	12.5 (15.2)	22.6 (3.06)
